# Effect of Heterogeneous Investments on the Evolution of Cooperation in Spatial Public Goods Game

**DOI:** 10.1371/journal.pone.0120317

**Published:** 2015-03-17

**Authors:** Keke Huang, Tao Wang, Yuan Cheng, Xiaoping Zheng

**Affiliations:** 1 Department of Automation, Tsinghua University, Beijing, 100084, China; 2 School of Mechanical and Electrical Engineering, Beijing University of Chemical Technology, Beijing, 100029, China; Hong Kong Baptist University, CHINA

## Abstract

Understanding the emergence of cooperation in spatial public goods game remains a grand challenge across disciplines. In most previous studies, it is assumed that the investments of all the cooperators are identical, and often equal to 1. However, it is worth mentioning that players are diverse and heterogeneous when choosing actions in the rapidly developing modern society and researchers have shown more interest to the heterogeneity of players recently. For modeling the heterogeneous players without loss of generality, it is assumed in this work that the investment of a cooperator is a random variable with uniform distribution, the mean value of which is equal to 1. The results of extensive numerical simulations convincingly indicate that heterogeneous investments can promote cooperation. Specifically, a large value of the variance of the random variable can decrease the two critical values for the result of behavioral evolution effectively. Moreover, the larger the variance is, the better the promotion effect will be. In addition, this article has discussed the impact of heterogeneous investments when the coevolution of both strategy and investment is taken into account. Comparing the promotion effect of coevolution of strategy and investment with that of strategy imitation only, we can conclude that the coevolution of strategy and investment decreases the asymptotic fraction of cooperators by weakening the heterogeneity of investments, which further demonstrates that heterogeneous investments can promote cooperation in spatial public goods game.

## Introduction

Cooperation and defection are two fundamental strategies for individuals confronted with every social dilemma [1–3]. According to Darwin’s theory of the origin of species, cooperation extinction is inevitable [4]. However, in the daily life, cooperation is abundantly observed in animal and human societies. One primary challenge in fields ranging from genetics and cell biology to evolutionary anthropology and behavioral economics is to explain the mechanism for these universal cooperative phenomena. Among the game theoretical approaches to investigating evolution of cooperation, one of the most popular frameworks to study the conflict between individuals is the so-called public goods game (PGG), in which several players decide whether or not to contribute to the common pool simultaneously [5–7]. In the model of PGG, cooperators contribute a fixed share to the public whereas defectors do not. Subsequently, all contributions are added up and multiplied by an enhancement factor *r*(*r* > 1), then the total welfare is equally divided among all players irrespective of their strategies. Consequently, players are faced with the temptation of being free-riders and rational players invest nothing which causes the “Tragedy of the Commons” [8–10].

Aiming at solving the above tragedy and investigating the reason why cooperators can survive in reality, several mechanisms have been proposed, such as reward [11], punishment [12–16], reputation [12, 17, 18], volunteering [9, 19], migration[20–22], social diversity [23–25] and so on. Besides, the enhancement factor evolving with time is identified as a useful mechanism for evolution of cooperative behavior in PGG [26]. Recently, the topology of the game has been intensively studied as a paradigm for illustrating how cooperative behavior evolves in spatial PGG [27–29], such as scale free networks [30–32], interdependent networks [33–39], and they have proved valuable for solving the tragedy to some extent. To summarize, in all the works above it is assumed that the investments of all the cooperators are identical, often equal to 1. However, it is worth mentioning that in the rapidly developing modern society, players are diverse and they often treat the same problem differently [40–42].

In the present work, the diversity of players is taken into consideration. With the aim of modeling the heterogeneous players in spatial PGG without loss of generality, it is assumed that the investment of a cooperator is a random variable with uniform distribution [1−*σ*,1+*σ*], the mean value of which is equal to 1. Moreover, we will discuss the impact of *σ* on evolution of cooperative behavior. In order to explore how heterogeneous investments affect the evolution of cooperative behavior, we compare the traditional update rule with the coevolution of strategy and investment, and by statistical analysis we further reveal that heterogeneous investments can promote cooperation in spatial PGG.

## Methods

In this article, the public goods game is considered on a two dimensional square lattice of size *L*×*L* with periodic boundary conditions. At the first step, each player can choose a pure strategy cooperation or defection randomly. Suppose that cooperator *x* contributes *Y*_*x*_ to the public goods while defectors contribute nothing, here *Y*_*x*_ is a random variable, and *Y*_*x*_ ∼ *U*[1−*σ*,1+*σ*],(0≤*σ*≤1). It is apparent that the mean of *Y*_*x*_ is 1 without loss of generality. Practically, when *σ* = 0, the model is identical with the traditional PGG. Suppose that the contribution of *x* is independent of that of other players.

For the sake of accelerating the simulations but without causing major modifications in the system’s dynamics, it is assumed that the score of player *x* is determined by a single PGG which involves the focal player and its four nearest neighbors [9]. The sum of all contributions in the group is multiplied by the factor *r*, and the resulting public goods are distributed among all the group members. Correspondingly, the payoff of player *x* is

$$P_{x}=r\frac{\sum_{y=1:N}Y_{y}}{N}-Y_{x}=\left\{ \begin{array}{ll} r\frac{\sum_{s_{y}=C}Y_{y}}{N}-Y_{x} & \text{if}\;s_{x}=C\\ r\frac{\sum_{s_{y}=C}Y_{y}}{N} & \text{if}\;s_{x}=D \end{array}\right.$$ (1)

where *s*_*x*_ denotes the strategy of player *x*, *N* denotes the group size, and in this article *N* = 5.

The Monte Carlo (MC) simulations start from a random initial strategy distribution, and the evolution procedure is subsequently controlled by a random sequential strategy update. In detail, player *x* and one of its neighbors *y* are chosen randomly. Following the payoffs *P*_*x*_,*P*_*y*_ calculated as described above, player *x* imitates the strategy of player *y* with a probability

$$p\left(s_{y}\to s_{x}\right)=\frac{1}{1+\exp\left[\left(P_{x}-P_{y}+\tau \right)/K\right]}$$ (2)

Where *τ* > 0 denotes the cost of strategy change and *K* introduces some noise to allow for irrational decisions, and in this article *τ* = *K* = 0.1 [9]. For the sake of comparison, the coevolution of strategy and investment, namely player *x* imitates also the investment of player *y* simultaneously, is considered. Furthermore, for the results presented in the next section, the square lattice used is sized *L* = 200 (In order to test whether the main results are robust against population size, this article had verified the results for different population sizes, and it is concluded that the main results are robust to the population size *L*). The asymptotic fraction of cooperators is determined by averaging over 5000 generations after a transient time of 55000 MCS.

## Results

**Fig 1:**
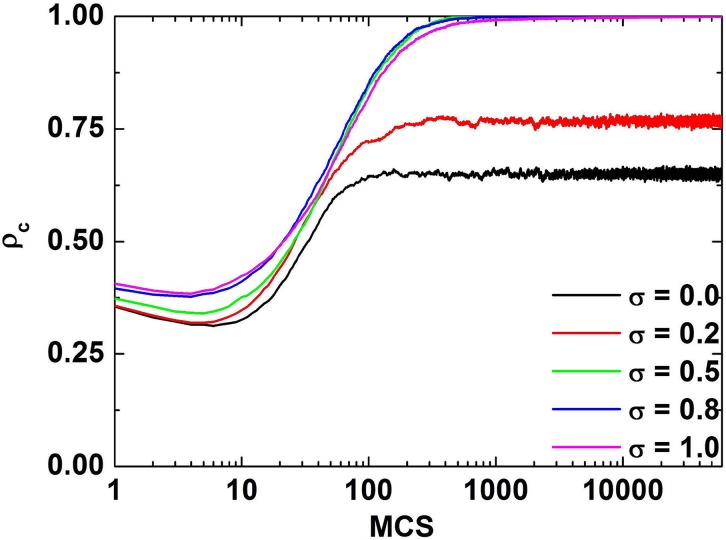
Time series of the fraction of cooperators for different values of *σ* when *r* equals to 5. The value of *σ* is equal to 0.0,0.2,0.5,0.8,1.0 respectively. While *σ* = 0, which is exactly the traditional case, the investment of all the cooperators is 1. With the increment of *σ*, the asymptotic fraction of cooperators will increase.

Firstly, it is instructive to examine the time series of the fraction of cooperators. Results presented in Fig. 1 are the time series of the fraction of cooperators for different values of *σ* when *r* equals to 5. The figure depicts that the fraction of cooperators decreases at the beginning of the evolution but increases to the value in the steady state later. The random distribution of the initial strategy gives rise to the decline of cooperation in the beginning, that is, the cooperators are dispersed and the defectors can invade the cooperators easily, which is corresponding to the initial phase in Fig. 1. As the evolution proceeds, the lived cooperators form clusters and the payoffs of them are enhanced, which restrain the invasion of the defectors. Meanwhile, the heterogeneous investments render the manifold payoff difference between cooperators and defectors, which in turn boosts the possibility for defectors to imitate cooperators.

**Fig 2:**
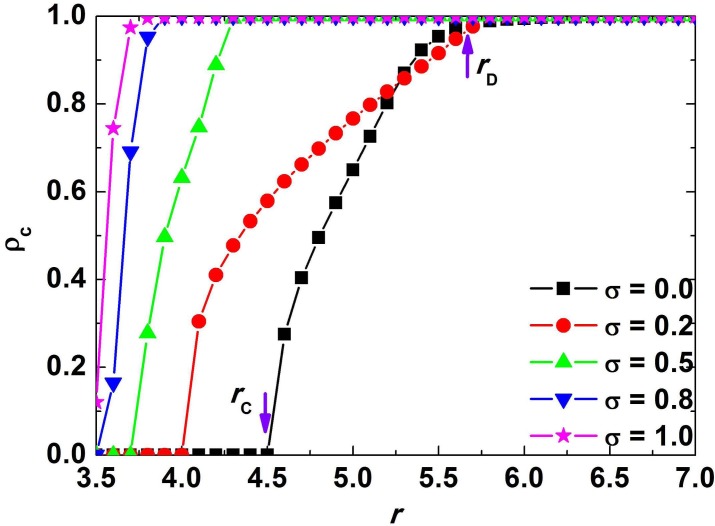
Relationship between asymptotic fraction of cooperators *ρ*_*C*_ and enhancement factor *r* corresponding to *σ* = 0, 0.2, 0.5, 0.8, 1.0 respectively. The curves in the figure show that the larger the value of *σ* is, the bigger the asymptotic fraction of cooperators *ρ*_*C*_ will be. In addition, larger value of *σ* can decrease the values of *r*_*C*_ and *r*_*D*_ effectively.

While Fig. 1 shows the time series of fraction of cooperators, the relationship between asymptotic fraction of cooperators *ρ*_*C*_ and enhancement factor *r* when *σ* = 0, 0.2, 0.5, 0.8, 1.0 is presented in Fig. 2. It is worth to note that, the black curve in Fig. 2 corresponding to *σ* = 0 is in agreement with the results in reference [9]. The asymptotic fraction of cooperators *ρ*_*C*_ depending on the enhancement factor *r* reveals that *ρ*_*C*_ is enhanced with increasing of *r* for different values of *σ*. Meanwhile, the larger the value of *σ* is, the bigger the asymptotic fraction of cooperators *ρ*_*C*_ will be. Besides, a large value of *σ* will guarantee that the cooperators will survive when the enhancement factor *r* is small. In addition, for each curve in Fig. 2, there exit two thresholds *r*_*C*_ and *r*_*D*_. In other words, if *r*<*r*_*C*_ the phase of the population after behavioral evolution is pure defectors, and if *r*>*r*_*D*_ the phase of the population is pure cooperators, while in case of *r*_*C*_<*r*<*r*_*D*_ the population consist of not only cooperators but also defectors. Fig. 2 clearly demonstrates that larger value of *σ* can decrease the value of *r*_*C*_ and *r*_*D*_ effectively. Therefore, to sum up, when the investment of a cooperator is a random variable with uniform distribution [1−*σ*,1+*σ*], the asymptotic fraction of cooperators can be promoted. Furthermore, the larger the value of *σ* is, the better the promotion effect will be.

**Fig 3:**
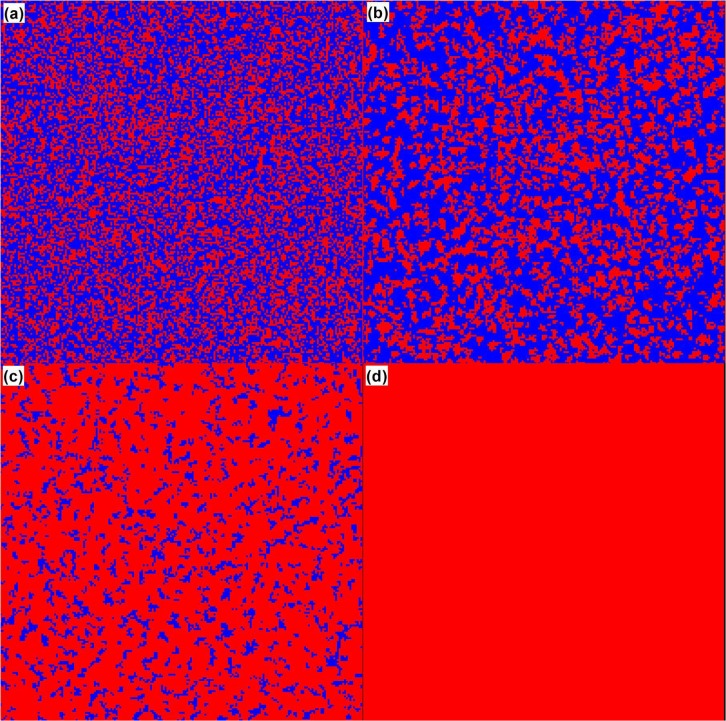
Typical snapshots of strategy distributions on the square lattice when *σ* = 0.5 and *r* = 5. Cooperators and defectors are colored red and blue, and the MCS of (a)-(d) is 1, 10, 100, 50000 respectively. The figure shows that the fraction of cooperators decreases at the beginning of the evolution, but as the evolution proceeds, the cooperators form into clusters to restrain the invasion of the defectors, and spread to the defectors reversely. At the end of the evolution all the players in the population hold the cooperation strategy.

In order to depict the evolutionary process vividly, the typical snapshots are represented in Fig. 3 when *σ* = 0.5 and *r* = 5. From the figure it can be clearly concluded that the fraction of cooperators decreases at the beginning of the evolution, but as the evolution proceeds, the cooperators form into clusters to restrain the invasion of the defectors and spread to the defectors reversely. At the end of the evolution all the players in the population hold the cooperation strategy.

**Fig 4:**
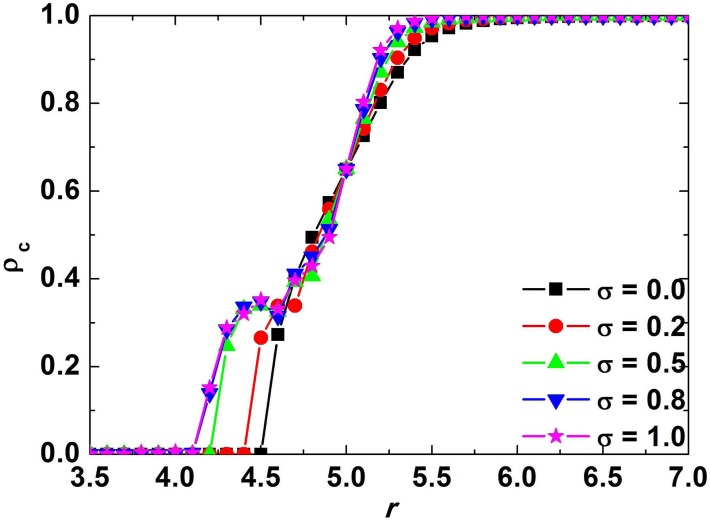
Relationship between asymptotic fraction of cooperators *ρ*_*C*_ and enhancement factor *r* corresponding to *σ* = 0, 0.2, 0.5, 0.8, 1.0 respectively when the coevolution of strategy and investment is taken into account. The curves show that if *σ*>0, the thresholds *r*_*C*_ and *r*_*D*_ decrease compared to the case of *σ* = 0, which is exactly the traditional situation. This implies that cooperation can be promoted by introducing the coevolution of strategy and investment.

Next, the relationship between fraction of cooperators and enhancement factor when the coevolution of strategy and investment is taken into consideration will be discussed. The curves in Fig. 4 show that if *σ*>0, the thresholds *r*_*C*_ and *r*_*D*_ become smaller compared to the case of *σ* = 0, which is exactly the traditional situation. This implies that cooperation can be promoted through the coevolution of strategy and investment.

**Fig 5:**
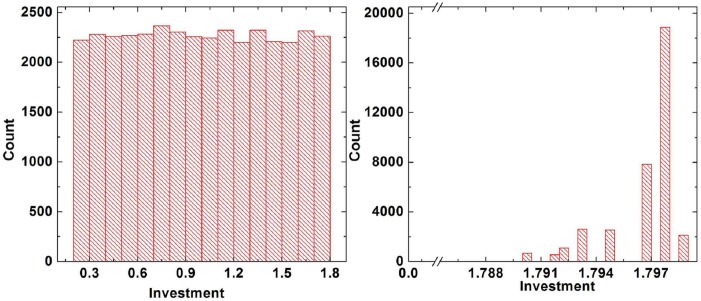
The steady distribution of investment corresponding to strategy evolution only (Left panel) and that corresponding to coevolution of strategy and investment (Right panel), when *σ* = 0.8, *r* = 5.2. From the figure, it can be found that when only strategy is updated, the distribution of the investment in steady state is still a uniform distribution [0.2,1.8], however, when the coevolution of strategy and investment is taken into account, the previous uniform distribution is severely distorted, and the steady investment satisfies a discrete distribution valued [1.786,1.790,1.792,1.793,1.795,1.797,1.798,1.799]. In conclusion, compared to the former case, as the heterogeneity of investment is weakened, the asymptotic fraction of cooperators *ρ*_*C*_ decreases in the case of coevolution.

Comparing the curves in Figs. 2 and 4 leads to the interesting conclusion that the promotion effect of coevolution of strategy and investment is inferior to that of strategy imitation only. With the aim to explore the difference between them, we will discuss the investment distribution of cooperators in steady state. Fig. 5 shows the investment distribution in case of coevolution of strategy and investment (Right panel) and the distribution in case of strategy imitation only (Left panel), and the parameters in the two situations are equal, namely *σ* = 0.8, *r* = 5.2. When only strategy evolves with time, the distribution of the investment in steady state is still a uniform distribution [0.2,1.8], which is the same with initial distribution. However, when the coevolution of both strategy and investment is considered, the distribution of investment after evolution comes to an end is severely distorted compared with the uniform distribution at the initial step, and the steady investment satisfies a discrete distribution valued [1.786,1.790,1.792,1.793,1.795,1.797,1.798,1.799]. Therefore, the heterogeneity of investment is weakened in the case of coevolution, leading to the decrement of asymptotic fraction of cooperators *ρ*_*C*_. In the same way, through statistical analysis it can be concluded that for the same enhancement *r*, the smaller the value of *σ* is, the more severe the distortion from previous uniform distribution will be. This implies that the coevolution of strategy and investment decreases the asymptotic fraction of cooperators *ρ*_*C*_ by weakening the heterogeneity of investment once again.

## Conclusions

To summarize, this article further explores the impact of heterogeneous investments on the evolution of cooperative behavior in spatial PGG. Above all, the relationship between asymptotic fraction of cooperators *ρ*_*C*_ and enhancement factor *r* when *σ* varies has been presented, and it can be concluded that the larger value of *σ* has the capacity to decrease the values of *r*_*C*_ and *r*_*D*_ effectively, and that the larger the value of *σ* is, the better the promotion effect will be. In addition, this article has also discussed the influence of heterogeneous investments when the coevolution of strategy and investment is taken into consideration. Comparing the promotion effect of coevolution of strategy and investment with that of strategy imitation only, the conclusion can be naturally drawn that the coevolution of strategy and investment decreases the asymptotic fraction of cooperators *ρ*_*C*_ by weakening the heterogeneity of investment. Therefore, the results above further demonstrate that heterogeneous investments are capable of promoting cooperation in spatial PGG.

## Supporting Information

S1 Dataset The dataset of Fig. 1. (XLSX)

S2 Dataset The dataset of Fig. 2. (XLSX)

S3 Dataset The dataset of Fig. 3. (XLSX)

S4 Dataset The dataset of Fig. 4. (XLSX)

S5 Dataset The dataset of Fig. 5. (XLSX)

## References

[1] Kollock P. Social dilemmas: The anatomy of cooperation. Annual review of sociology. 1998:183–214.

[2] Fehr E, Fischbacher U. Social norms and human cooperation. Trends in cognitive sciences. 2004;8(4):185–90. 15050515 10.1016/j.tics.2004.02.007

[3] Bornstein G, Ben-Yossef M. Cooperation in intergroup and single-group social dilemmas. Journal of Experimental Social Psychology. 1994;30(1):52–67.

[4] Pennisi E. How did cooperative behavior evolve? Science. 2005;309(5731):93–. 15994539 10.1126/science.309.5731.93

[5] Szabó G, Fath G. Evolutionary games on graphs. Physics Reports. 2007;446(4):97–216.

[6] Perc M, Gómez-Gardeñes J, Szolnoki A, Floría LM, Moreno Y. Evolutionary dynamics of group interactions on structured populations: a review. Journal of The Royal Society Interface. 2013;10(80):20120997 10.1098/rsif.2012.0997 23303223 PMC3565747

[7] Perc M, Grigolini P. Collective behavior and evolutionary games–An introduction. Chaos, Solitons & Fractals. 2013;56:1–5.

[8] Killingback T, Doebeli M, Hauert C. Diversity of cooperation in the tragedy of the commons. Biol Theory. 2010;5:3–6.

[9] Szabó G, Hauert C. Phase transitions and volunteering in spatial public goods games. Physical Review Letters. 2002;89(11):118101 12225171 10.1103/PhysRevLett.89.118101

[10] Szolnoki A, Perc M, Szabó G. Topology-independent impact of noise on cooperation in spatial public goods games. Physical Review E. 2009;80(5):056109. 10.1103/PhysRevE.80.056109 20365045

[11] Szolnoki A, Perc M. Reward and cooperation in the spatial public goods game. EPL (Europhysics Letters). 2010;92(3):38003.

[12] Brandt H, Hauert C, Sigmund K. Punishment and reputation in spatial public goods games. Proceedings of the Royal Society of London Series B: Biological Sciences. 2003;270(1519):1099–104. 12803901 10.1098/rspb.2003.2336 PMC1691345

[13] Helbing D, Szolnoki A, Perc M, Szabó G. Punish, but not too hard: how costly punishment spreads in the spatial public goods game. New Journal of Physics. 2010;12(8):083005.

[14] Brandt H, Hauert C, Sigmund K. Punishing and abstaining for public goods. Proceedings of the National Academy of Sciences. 2006;103(2):495–7. 16387857 10.1073/pnas.0507229103 PMC3020126

[15] Fehr E, Gächter S. Cooperation and punishment in public goods experiments. American Economic Review. 2000:980–94.

[16] Wang Z, Xia C-Y, Meloni S, Zhou C-S, Moreno Y. Impact of social punishment on cooperative behavior in complex networks. Scientific reports. 2013;3. 10.1038/srep03055 PMC3808815 24162105

[17] Milinski M, Semmann D, Krambeck H-J. Reputation helps solve the ‘tragedy of the commons’. Nature. 2002;415(6870):424–6. 11807552 10.1038/415424a

[18] Wang Z, Wang L, Yin Z-Y, Xia C-Y. Inferring reputation promotes the evolution of cooperation in spatial social dilemma games. PLoS One. 2012;7(7):e40218 10.1371/journal.pone.0040218 22808120 PMC3392274

[19] Semmann D, Krambeck H-J, Milinski M. Volunteering leads to rock–paper–scissors dynamics in a public goods game. Nature. 2003;425(6956):390–3. 14508487 10.1038/nature01986

[20] Wang L, Wang Z, Zhang Y, Li X. How human location-specific contact patterns impact spatial transmission between populations? Scientific reports. 2013;3. 10.1038/srep01468 PMC3601479 23511929

[21] Wang Z, Szolnoki A, Perc M. If players are sparse social dilemmas are too: Importance of percolation for evolution of cooperation. Scientific reports. 2012;2. 10.1038/srep00369 PMC3328045 22511999

[22] Wang Z, Du W-B, Cao X-B, Zhang L-Z. Integrating neighborhoods in the evaluation of fitness promotes cooperation in the spatial prisoner’s dilemma game. Physica A: Statistical Mechanics and its Applications. 2011;390(7):1234–9.

[23] Santos FC, Santos MD, Pacheco JM. Social diversity promotes the emergence of cooperation in public goods games. Nature. 2008;454(7201):213–6. 10.1038/nature06940 18615084

[24] Wang Z, Perc M. Aspiring to the fittest and promotion of cooperation in the prisoner’s dilemma game. Physical Review E. 2010;82(2):021115. 10.1103/PhysRevE.82.021115 20866783

[25] Wang Z, Murks A, Du W-B, Rong Z-H, Perc M. Coveting thy neighbors fitness as a means to resolve social dilemmas. Journal of theoretical biology. 2011;277(1):19–26. 10.1016/j.jtbi.2011.02.016 21354430

[26] Chen X, Liu Y, Zhou Y, Wang L, Perc M. Adaptive and bounded investment returns promote cooperation in spatial public goods games. PloS one. 2012;7(5):e36895 10.1371/journal.pone.0036895 22615836 PMC3353963

[27] Gómez-Gardeñes J, Romance M, Criado R, Vilone D, Sánchez A. Evolutionary games defined at the network mesoscale: The public goods game. Chaos: An Interdisciplinary Journal of Nonlinear Science. 2011;21(1):016113. 10.1063/1.3535579 21456855

[28] Zhang G-Q, Sun Q-B, Wang L. Noise-induced enhancement of network reciprocity in social dilemmas. Chaos, Solitons & Fractals. 2013;51:31–5.

[29] Wang L, Li X. Spatial epidemiology of networked metapopulation: An overview. Chinese Science Bulletin. 2014;59(28):3511–22. 10.1007/s11434-014-0499-8 PMC7088704 32214746

[30] Rong Z, Wu Z-X. Effect of the degree correlation in public goods game on scale-free networks. EPL (Europhysics Letters). 2009;87(3):30001.

[31] Guan J-Y, Wu Z-X, Wang Y-H. Effects of inhomogeneous activity of players and noise on cooperation in spatial public goods games. Physical Review E. 2007;76(5):056101 18233712 10.1103/PhysRevE.76.056101

[32] Yuan W-J, Zhou J-F, Li Q, Chen D-B, Wang Z. Spontaneous scale-free structure in adaptive networks with synchronously dynamical linking. Physical Review E. 2013;88(2):022818 24032894 10.1103/PhysRevE.88.022818

[33] Wang Z, Szolnoki A, Perc M. Interdependent network reciprocity in evolutionary games. Scientific reports. 2013;3. 10.1038/srep01183 PMC3560361 23378915

[34] Wang Z, Wang L, Perc M. Degree mixing in multilayer networks impedes the evolution of cooperation. Physical Review E. 2014;89(5):052813 25353850 10.1103/PhysRevE.89.052813

[35] Boccaletti S, Bianconi G, Criado R, Del Genio C, Gómez-Garde?es J, Romance M, et al The structure and dynamics of multilayer networks. Physics Reports. 2014;544(1):1–122. 10.1016/j.physrep.2014.07.001 PMC7332224 32834429

[36] Wang Z, Szolnoki A, Perc M. Self-organization towards optimally interdependent networks by means of coevolution. New Journal of Physics. 2014;16(3):033041.

[37] Wang Z, Szolnoki A, Perc M. Optimal interdependence between networks for the evolution of cooperation. Scientific reports. 2013;3. 10.1038/srep02470 PMC3747507 23959086

[38] Zhao D, Wang L, Li S, Wang Z, Wang L, Gao B. Immunization of Epidemics in Multiplex Networks. PloS one. 2014;9(11):e112018 10.1371/journal.pone.0112018 25401755 PMC4234317

[39] Wang Z, Szolnoki A, Perc M. Evolution of public cooperation on interdependent networks: The impact of biased utility functions. EPL (Europhysics Letters). 2012;97(4):48001.

[40] Burlando RM, Guala F. Heterogeneous agents in public goods experiments. Experimental Economics. 2005;8(1):35–54.

[41] Shi D-M, Zhuang Y, Wang B-H. Group diversity promotes cooperation in the spatial public goods game. EPL (Europhysics Letters). 2010;90(5):58003.

[42] Fischbacher U, Gächter S. Heterogeneous social preference and the dynamics of free riding in public goods. CeDEx Discussion Paper, The University of Nottingham, 2006.

